# Conditional cash transfer interventions to support syphilis treatment in vulnerable populations: a quasi-experimental study among displaced and host communities in a border city of Colombia

**DOI:** 10.1016/j.lana.2025.101301

**Published:** 2025-11-14

**Authors:** Merike Blofield, Andrea L. Wirtz, Magaly Pedraza, Rafael Olarte, Doris Parada

**Affiliations:** a German Institute for Global and Area Studies, Hamburg, Germany; b University of Hamburg, Hamburg, Germany; c Johns Hopkins University, Baltimore, MD, USA; d Universidad Francisco de Paula Santander, Cúcuta, Colombia; e Hospital Universitario Erasmo Meoz, Cúcuta, Colombia

**Keywords:** Infectious diseases, Sexually transmitted infections, Syphilis, Colombia, Venezuela, Displaced populations, Host communities, Conditional cash transfers, Quasi-experiment, Diagnosis, Treatment adherence, Vulnerable Populations, Penicillin

## Abstract

**Background:**

Syphilis incidence is increasing globally; however, cost and time are significant barriers to treatment completion rates among vulnerable populations, including displaced populations and host communities. To inform public health strategies, we aimed to test whether conditional cash transfers (CCT) increased completion of syphilis treatment among a community sample in a border city of Colombia.

**Methods:**

We embedded a quasi-experimental trial of a CCT intervention in a community sexual health program serving participants aged 14 years and older in low-income settlements around Cúcuta, Colombia in 2023. The program included workshops and syphilis screening. Individuals with laboratory-confirmed syphilis were eligible for inclusion in the trial. Both control and CCT arms provided syphilis diagnostics, counseling and free treatment. CCT consisted of cash payments of USD$12.69 for completion of each of two follow-up treatments. We used generalized linear models to estimate the effect of CCT on treatment completion, defined as three doses of penicillin.

**Findings:**

Of 1751 workshop participants, 114 had laboratory-confirmed syphilis and were enrolled in the trial. Participants were 56% female (64/114) and 44% male (50/114), with 6 participants (5.3%) identifying as transgender, regardless of sex at birth. Participants included 47% Venezuelan migrants staying in Cúcuta (53/114), 26% Colombian returnees (30/114), and 19% Colombians from the host community (22/114). Data on ethnicity was not collected. Median age was 34.5 years (IQR: 25.0–46.0). More than three-quarters (78%, 39/50) of CCT participants completed the three-dose treatment regimen compared to 45% (29/64) of control participants, a risk difference of 33% (p < 0.001). In adjusted models, CCT-assigned participants had a 36% higher treatment completion rate compared to control-assigned participants (adjusted risk difference: aRD: 0.36, 95% CI: 0.19–0.53).

**Interpretation:**

Conditional cash transfers might enhance syphilis treatment adherence among populations facing socioeconomic challenges.

**Funding:**

German Development Cooperation Agency, 10.13039/501100011099Deutsche Gesellschaft für Internationale Zusammenarbeit.

**Research in context**

**Evidence before this study**

Diagnosis and treatment are fundamental to preventing long term health sequelae of untreated syphilis and congenital syphilis, as well as to preventing onward transmission.

We searched PUBMED and Google Scholar in August 2024 and during revisions in April 2025 for studies on syphilis treatment adherence, using the terms “syphilis treatment adherence”, “syphilis treatment uptake”, “cash transfers and syphilis”, “cash transfers and sexually transmitted infections” and “cash transfers and treatment adherence”. Little research exists to describe treatment uptake or adherence among adult populations outside of prenatal care settings. Three clinical cohorts have demonstrated variable treatment completion among people diagnosed with syphilis. To our knowledge, no interventions have been tested to improve syphilis treatment adherence among socially vulnerable and displaced people.

A large body of literature has examined the effects of conditional and unconditional cash transfers on behavioural changes and improved health outcomes among vulnerable populations. Two systematic reviews on conditional cash transfers and HIV outcomes have reported generally positive, though some mixed results, predominantly in Sub Saharan Africa. Findings, however, may be related to the many confounding factors between transfers and the outcome of interest. It is possible that cash transfer interventions may be more effective for supporting adherence to treatment of limited durations, such as syphilis, compared to long-term treatment for HIV. However, to our knowledge, there have been no evaluations of the use of cash transfer interventions to improve syphilis treatment adherence.

**Added value of this study**

This quasi-experimental study trialed the use of a conditional cash transfer (CCT) intervention to promote the completion of syphilis treatment, defined as 3 doses of penicillin. Set in a border city of Colombia, it included a sample of people (n=114) with laboratory-confirmed syphilis infection. Participants were drawn from a broader group of people displaced from Venezuela and members of the host community who were recruited in low-income settings to participate in community health workshops (N=1750). Compared to a control group that provided syphilis diagnostics, counseling and free treatment, participants assigned to a CCT were nearly twice as likely to complete treatment compared to those in the control group. Notably, the majority of participants in both CCT and control groups completed at least one dose of treatment, which would be sufficient for early syphilis infections, according to the WHO; however, the CCT demonstrated improvements in completing the repeated injections over time.

**Implications of all the available evidence**

This study adds to the growing literature that finds that combining social protections such as cash transfers with public health and medical interventions can improve uptake of healthcare interventions. Conditional cash transfer interventions may be particularly effective for treatments of limited duration such as syphilis and among socially vulnerable populations, such as communities with a high share of displaced people. Further, this study highlights the feasibility of integration of displaced people into sexual health interventions with host communities. Using cash transfers to support healthcare is especially promising in the Americas given their broad use and acceptability in the region, and their potential cost-effectiveness when compared to the social and medical costs of long-term health sequelae of untreated syphilis and congenital syphilis.

## Introduction

The global burden of syphilis has expanded over the last three decades, with the greatest increase in age-standardised incidence rates in the Americas.^1^^,^^2^ As of 2016, the Pan American Health Organisation (PAHO) reported the prevalence of syphilis in men was 0.91%, 0.92% in women, and 0.86% among pregnant women in Latin America.^3^ In Colombia, national-level data from 2016 estimated a 1.3% prevalence among the general adult population.^4^ The incidence of congenital syphilis in Colombia was 2.4 per 1000 live and stillbirths in 2023 and 5.7 per 1000 live births in 2022 in the North of Santander, the border region with Venezuela.^5^

While public health surveillance has provided population prevalence estimates^1^, ^2^, ^3^ and multiple studies have documented the increased prevalence of syphilis during pregnancy and congenital syphilis,^3^^,^^5^ less is known about the experiences of displaced populations -populations that are increasing in the region due to the political crisis in Venezuela and historical internal conflict in Colombia. The causes and contexts of displacement create social vulnerabilities that place people at increased risk for disease, including syphilis, due to cost and other barriers in access to prevention, diagnosis, and treatment.^6^ A recent study of the population prevalence of syphilis infection among Venezuelan migrants residing in urban settings of Colombia in 2021-22 was estimated at 5.1% (95% CI: 4.6–5.6) and as high as 9.0% (95% CI 5.4–14.6) among pregnant women.^7^ In Colombia, syphilis diagnosis and treatment are covered through national health insurance schemes, but are costly for migrants with an irregular status and other returned Colombians who are not affiliated with the health system. Extraneous costs associated with transportation and time away from work serve as additional barriers to STI services.

Diagnosis and treatment are fundamental to preventing long-term health sequelae of untreated syphilis and congenital syphilis, as well as to preventing onward transmission. Untreated syphilis can lead to severe neurologic, cardiovascular, and ophthalmic complications.^8^ Less research exists to describe treatment adherence among adult populations outside of prenatal care settings in the region; however, the few that exist point to variable treatment completion among people diagnosed with syphilis. Among patients diagnosed with syphilis, three clinical cohorts, in the United States, Argentina and Chile, reported treatment completion rates that ranged from 52 to 78% across the three studies.^9^, ^10^, ^11^ Moreover, the study in Chile reported that 88% of women completed treatment compared to 65% of male partners, thus underscoring the need for better strategies to increase completion rates across subpopulations.^10^ Qualitative research among women who delivered newborns with congenital syphilis in Cúcuta, a border city of Colombia, described the difficulty of screening and treating intimate partners for syphilis, particularly partners who remained in Venezuela, which resulted in risk of reinfection.^12^ As clinical samples of people seeking STI testing and treatment, these findings likely overestimate treatment completion rates among the general population, including those who are asymptomatic. Nonetheless, these findings indicate that ensuring the availability of treatment may be necessary but not sufficient to guarantee adherence to treatment.

The issue of adherence to syphilis treatment has received limited attention. A recent randomised trial among 64 postpartum women in Brazil demonstrated the benefit of an educational intervention vs. standard of care in improving knowledge about syphilis acquisition and treatment, and increased knowledge across both arms was found to be associated with improved treatment adherence.^13^ One protocol for a randomized three-arm trial to evaluate monitoring strategies to improve adherence to treatment in Brazil was published in 2022, though results have not yet been published.^14^ While education and monitoring are likely to support improved treatment adherence, they may fail to address structural barriers for more vulnerable communities related directly to cost of treatment and clinical services, as well as indirectly related to time, transportation, childcare, and lost wages spent accessing services, particularly for treatment that requires multiple clinical visits.^15^

Originating in the Americas, a growing body of literature has documented the positive impact of cash transfers for socially vulnerable populations, on numerous development goals, including poverty, health, education, and empowerment.^16^ These transfers can be conditional or unconditional. Unconditional transfers seek to alleviate poverty and provide social protection. Conditional cash transfers (CCTs) link the incentive to a specific behavioural outcome, such as attending a health clinic or remaining STI-free. CCTs were developed based on behavioural economic theory, which broadly posited that fully informed individuals may make decisions that appear to contradict their long-term future or health goals because of their current situation.^15^ Thus, incentives may encourage individuals to make decisions that improve long-term health goals. CCTs are also supported by psychological theories, specifically self-efficacy theory and self-determination theory. Investigational research related to self-efficacy theory suggest that positive incentives may promote self-motivation when they enhance or validate self-efficacy beliefs. Further, when individuals aim for and master a desired action, they feel satisfaction and increased self-efficacy for that particular action.^15^ Self-determination theory complementarily suggests that both intrinsic and extrinsic motivation is important for performing a health behaviour; thus, while individuals may have an individual desire to perform a health behaviour, an outside force or an external reward, such as a CCT, may be the driver to perform the task.^15^

In Latin America, national-level conditional cash transfer programs targeting low-income families with children provide small cash transfers to families conditional on children attending school and getting health check-ups. Studies on the largest program of its kind in the region, the Brazilian Bolsa Familia, show that the program is also associated with health benefits for adult recipients, through both alleviation of poverty-related risk factors and access to healthcare. Two analyses found that Bolsa Família was associated with significant reduction in AIDS incidence, morbidity and mortality.^17^^,^^18^ A quasi-experimental study of Bolsa Familia recipients found that being a recipient of the program was associated with greater leprosy multidrug therapy adherence.^19^

Cash transfer programs have been used at a smaller scale to target specific behaviours and outcomes. Two systematic reviews have addressed the effects of cash transfers targeting HIV and STI prevention and care outcomes.^20^^,^^21^ One addressed HIV prevention and included studies that provided a cash transfer intervention, savings program, or program to reduce school costs from 27 different interventions or populations, predominantly from sub-Saharan Africa. The review found mixed and limited evidence for “large-scale impacts of cash transfers reducing HIV risk”, though it did not evaluate HIV or other STI prevention or treatment adherence.^20^ Another systematic review and meta-analysis of cash transfer interventions for HIV prevention and care found that cash transfer programs were associated with reduced HIV incidence and improved retention in HIV care for pregnant women.^21^ The meta-analysis of three studies in African settings found no effect of unconditional or CCTs in improving HIV treatment adherence, though two individual studies showed improvements in adherence associated with the interventions.^21^ It is possible that cash transfer interventions may be more effective for supporting adherence to treatment of limited durations, such as syphilis, compared to long-term treatment for HIV; however, to our knowledge, there have been no evaluations of the use of cash transfer interventions to improve syphilis treatment adherence.

We aimed to test the effect of CCTs on adherence to syphilis treatment in vulnerable displaced and host communities of the Colombia–Venezuela border. We hypothesised that CCTs would address the opportunity cost of time to seek treatment for individuals who have unmet basic needs and who are likely to prioritise daily survival for themselves and their families over treatment adherence. This opportunity cost may be especially high among non-pregnant individuals and in a context of high population mobility.

## Methods

### Study design and participants

We embedded a quasi-experimental study into a community health mobilisation pilot project that involved outreach to low-income groups within and around the city of Cúcuta. Cúcuta is located on the Venezuelan-Colombian border and known to host a large number of Venezuelan migrants and refugees and returned Colombians (“returnees”) who had previously sought refuge in Venezuela, alongside Colombians with no displacement history (i.e., host community). Cúcuta reflects the changing population distributions observed in border and non-border cities in Colombia and other Andean countries. As of November 2024, an estimated 7.89 million Venezuelans have been displaced from Venezuela as a result of a decade-long economic and political crisis; more than 6.71 million are estimated to have remained in the Latin American and Caribbean region, of whom 2.81 million, or about 42%, are residing in Colombia.^22^ In border areas, some Venezuelans-known as pendular migrants-move back and forth between Venezuela and Colombia for work or services, while others move with the intention of permanent stay. Further, Colombia hosts one of the largest populations of internally displaced people, with over 6.8 million people estimated to be internally displaced as of the end of 2022 and more than half a million Colombians who have returned to Colombia after previously seeking asylum in Venezuela.^23^

The project and study were implemented between March and August 2023 by CARE Colombia. Participants were recruited to 82 sexual health workshops in 38 communities, which were organised in collaboration with local community leaders. The workshops were open to everyone 14 years and older and included discussions of syphilis and the health impacts of untreated syphilis. Participants were provided with syphilis testing and treatment, as needed.

### Syphilis testing and treatment

Rapid treponemal tests for syphilis were provided to those who attended the workshops. Participants who had a preliminary positive rapid test were provided confirmatory testing using laboratory-performed non-treponemal VDRL testing. Participants with confirmed syphilis infection were provided treatment free of cost at a sub-contracted health clinic in central Cúcuta. Given syphilis of unknown duration or latent syphilis, individuals with confirmed syphilis were treated with three doses of 2.4 million units of benzathine penicillin G intramuscularly weekly, one week apart each, with the first dose administered immediately. Testing and treatment followed national guidelines.^24^ Telephone monitoring was also provided to all participants to encourage treatment adherence.

### Conditional cash transfer vs. control quasi-experimental trial

Eligibility criteria for inclusion in the quasi-experimental trial included laboratory confirmed syphilis infection and age ≥ 15 years. As a pilot intervention, the sample size was based on available resources. The intervention was allocated chronologically and was therefore non-randomised. Treatment allocation was divided into four phases, and individuals who met eligibility criteria were assigned to the CCT arm during phases one and three, until at least 50 individuals had been allocated to each arm (i.e., Phase one had 25 participants allocated to CCT arm, followed by Phase two in which 25 participants were allocated to control arm, and so forth). This sequential, non-randomised allocation was deemed appropriate due to ethical and practical reasons: random allocation within a group of workshop participants or who live in the same settlement could create resentment and frustration among those who were not assigned to the CCT arm. Sequential allocation reduced the likelihood that participants who were assigned to different treatments may know each other and, thus, reduce risk of selection bias.

Participants assigned to the CCT arm were informed that they would receive a cash transfer upon returning for and receiving the second and the third doses each. Cash transfer incentives were estimated to be equivalent to a day's labor at the national minimum wage plus the cost of public transportation and a meal (two transfers at $50,000 pesos [USD$12.69 in 2023]). Participants assigned to the control arm received only the syphilis testing, free treatment, and monitoring activities. Data on self-reported sex, sexual orientation, gender, nationality, marital status and education was collected. Data on ethnicity was not collected.

### Ethical review

Written informed consent was obtained from the participants prior to their participation. According to Colombian law (resolution 8430/1993) this type of study is classified as minimal risk. The same law allows adolescents with sufficient maturity to give informed consent. The organisation responsible for carrying out the health services pilot and the study, CARE Colombia, had protocols in place for the mitigation of risks inherent to provision of cash transfers. The ethics committee of the Universidad Francisco Paula de Santander reviewed and approved the execution of the study.

### Statistical analysis

We analysed descriptive statistics of sociodemographic characteristics of all 1751 participants in the community health mobilisation project (supplementary Material) and among the participants enrolled in the quasi-experimental trial.

Our primary outcome of interest was syphilis treatment completion, defined in this context as three injections of penicillin. We used generalised linear models for binomial outcomes with robust variance estimation to estimate the magnitude and strength of the association between CCT (vs. control) and completion of syphilis treatment. We use the generalised linear models to calculate unadjusted and adjusted risk differences (RD) and relative risks (RR). Due to the frequent nature of the outcome (60%) we calculated the RR and adjusted relative risks (aRR) of treatment completion, to avoid overestimation that occurs when estimating odds ratios for common outcomes.^25^ To address potential confounders in the quasi-experimental design, we used multivariate analysis to adjust for differences identified in the treatment arm or hypothesised to be associated with treatment completion, including age, nationality, sex, and education. Education was collapsed to none or primary vs. high school or higher to avoid sparse data bias. Because migration profiles and affiliation with health insurance are highly associated with nationality, we did not include these covariates in the models when adjusting for nationality and to avoid sparse data bias.^26^

We conducted a sensitivity analysis by using propensity score matching with a logistic treatment model and robust variance estimation to estimate the effect of CCT on treatment completion, calculated as the risk difference. In this analysis, the CCT was selected as the intervention variable, the outcome variable was specified as treatment completion and covariates to adjust for differences identified across treatment arms or hypothesised to be associated with treatment completion were included in the model. Covariates in Model 1 included nationality, age, and education status and covariates in Model 2 included sex, nationality, age, and education status. We performed a 1:1 matching between subjects who received CCT and controls based on the similarity of their propensity scores. Covariate balance was verified post-matching to ensure comparability between CCT and control arms^27^ by comparing standardised mean difference, variance ratios, and box plots of propensity scores before and after matching. Statistical analyses were conducted using Stata 17 (College Station, TX).

### Role of funding source

The funder of the study had no role in the study design, data collection, data analysis, data interpretation, or writing of the report. All authors had full access to all the anonymised data in the study and the corresponding author had final responsibility for the decision to submit for publication.

## Results

**Fig. 1:**
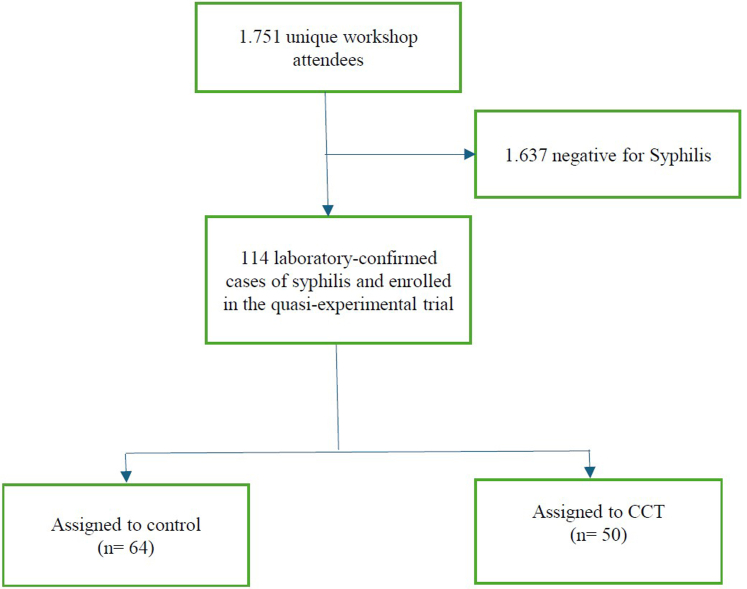
Flow diagram of quasi-experimental trial to test the effect of conditional cash transfer on the completion of a three-dose regimen for syphilis treatment.

A total of 1751 people participated in the community workshops. All 1751 workshop participants completed rapid tests for syphilis; of these, 114 (6.5%) were identified with laboratory-confirmed syphilis and were enrolled in the trial. The remainder of participants (n = 1637) tested negative for syphilis and were not eligibile for participation in the trial. In total, 50 participants were assigned to the CCT experiment (44% of trial participants; Fig. 1).

Trial participants were a median age of 34.5 years (IQR: 25.0–46.0) and slightly more than half (56%) were female. Participants were relatively evenly distributed by country of origin, with 45% (51/114) reporting Colombian nationality, 54% (62/114) reporting Venezuelan nationality, and 1 participant reporting other nationality. As a border area, only 19% (22/114) of the sample was comprised of the host Colombian population, while many had recently migrated to Colombia, including Colombians who had been previously displaced. Specifically, 26% (30/114) identified as Colombian returnees from Venezuela, 47% (53/114) identified as Venezuelan migrants with plans to stay in Colombia, and 8% (9/114) identified as migrants who were in transit or in a pendular situation (travel regularly between Colombia and Venezuela for work and other necessities). Only 23% (26/114) were affiliated with health insurance; however, this is largely associated with nationality, as Venezuelans must have a regular migration status before they can register for health insurance. Thus, only five Venezuelans were affiliated in this sample. Education was relatively low with only 49% (56/114) having high school or higher education. Sixty percent (68/144) of the sample completed syphilis treatment by the end of the study. Table 1 displays characteristics of the quasi-experimental trial participants, stratified by intervention arm. Participants appeared to be different across arms in terms of age and nationality. Supplementary Table S1 displays characteristics of community workshop and quasi-experimental trial participants.

**Table 1:** Characteristics of participants in a quasi-experimental trial to test the effect of conditional cash transfer on completion of a 3-dose regimen for syphilis treatment among people with syphilis diagnosis in a Colombian border city, 2023 (N = 114).

	Intervention				Total (N = 114)		p-value
	Control (n = 64)		Conditional cash transfer (n = 50)				
**Median age**, years (IRQ)	30.5	(22.5–39.5)	40.5	(29.0–52.0)	34.5	(25.0–46.0)	0.004
	**n**	**Col %**	**n**	**Col %**	**n**	**Col %**	**p-value**

**Sex**							0.243
Male	25	39.1	25	50.0	50	43.9	
Female	39	60.9	25	50.0	64	56.1	
**Gender and sexual orientation**							
Cisgender, gay	0	0	2	4.0	2	1.8	0.131
Cisgender, heterosexual	62	98.9	44	88.0	106	93.0	
Transgender, any sexual orientation	2	3.1	4	8.0	6	5.3	
**Nationality**							0.004
Colombian	21	32.8	30	60.0	51	44.7	
Venezuelan or other	43	67.2	20	40.0	63	55.3	
**Marital status**							0.050
Married or civil union	28	43.8	13	26.0	41	36.0	
Single or separated	36	56.2	37	74.0	73	64.0	
**Highest education started or completed**							0.556
None or primary	31	48.4	27	54.0	58	50.9	
Highschool or above	33	51.6	23	46.0	56	49.1	
**Treatment**							
**Number of doses completed**							<0.001
0	1	1.6	2	4.0	3	2.6	
1	16	25.0	7	14.0	23	20.2	
2	18	28.1	2	4.0	20	17.5	
3	29	45.3	39	78.0	68	59.6	
**Completed treatment (3 doses)**							<0.001
No	35	54.7	11	22.0	46	40.4	
Yes	29	45.3	39	78.0	68	59.6	

Overall, 78% (39/50) of CCT participants completed the three-dose regimen of treatment compared to 45% (29/64) in the control arm. Table 2 displays the estimated treatment effect as risk differences associated with the intervention in the unadjusted model and co-variate adjusted models. In the unadjusted model, CCT was associated with a 33% increase in treatment completion when compared to control (RD: 0.337, 95% CI: 0.15–0.50). The magnitude of association strengthened slightly when adjusting for other covariates (Model 2: aRD: 0.36, 95% CI: 0.19–0.53). Supplementary Table S2 displays the corresponding relative risks in the estimated models. No other covariates were associated with treatment completion in adjusted models.

**Table 2:** Estimated risk difference of the effect of CCT compared to control on the completion of treatment of syphilis in a sample of people with syphilis diagnosis in a border city of Colombia, 2023 (N = 114).

Characteristic	Model 1				Model 2				Model 3				Model 4			
	RD	95% CI		p-value	aRD	95% CI		p-value	aRD	95% CI		p-value	aRD	95% CI		p-value
CCT (vs. Control)	0.33	0.15	0.50	p < 0.001	0.36	0.19	0.53	p < 0.001	0.36	0.20	0.52	p < 0.001	0.35	0.18	0.52	p < 0.001
Age (continuous)					−0.001	−0.007	0.004	0.538	−0.001	−0.008	0.003	0.581	−0.001	−0.007	0.004	0.590
Nationality: Venezuelan or other (vs. Colombian)					0.043	−0.13	0.22	0.624	0.0048	−0.12	0.22	0.581	0.019	−0.16	0.20	0.840
Female sex (vs. Male)					–	–	–	–	–	–	–	–	0.69	−0.10	0.24	0.431
Education: Highschool or above (vs. none or primary)					–	–	–	–	−0.035	−0.20	0.13	0.672	–	–	–	–

Notes: Completed treatment is defined as received three doses of benzathine penicillin G intramuscularly weekly; RD risk difference; aRD: adjusted risk difference calculated from model that includes listed co-variates (i.e., Model 2 estimates the effect of CCT vs. control, adjusting for age and nationality); RDs and aRDs were calculated using generalised linear models for binomial outcomes with robust variance estimation.

Among participants assigned to the CCT, there was no statistical difference in participant characteristics when comparing those with incomplete and complete treatment. We did observe that higher proportions of Venezuelans (compared to Colombians), participants without health insurance (compared to those with insurance), and married people (compared to single) completed the treatment regimen (Supplementary Table S3).

In the sensitivity analysis, which estimated the treatment effects using propensity score matching by age, nationality, and education, there was a 29% increase in treatment completion comparing CCT to control (aRD: 0.29, 95% CI: 0.069–0.49, p = 0.005). We observed similar results when sex was added to the model (aRD: 0.29; 95% CI: 0.11–0.46, p = 0.002). Evaluation of standardised mean differences, variance ratios, and box plots of propensity scores demonstrated appropriate balance in the matched sample (Supplementary Tables S4 and S5; Figures S1, S2).

## Discussion

This quasi-experimental study trialed the use of a conditional cash transfer intervention in a border city of Colombia to promote the completion of syphilis treatment, defined as three doses of penicillin, among 114 Venezuelans and Colombians living in vulnerable communities. Compared to a control group that was provided with free syphilis diagnostics, counseling and treatment, CCT in combination with those services was found to be associated with a 36% improvement in treatment completion. These findings suggest that CCTs in the context of a community health mobilisation project might enhance treatment adherence among populations facing socioeconomic challenges.

Notably, the majority of participants in both CCT and control groups completed at least one dose of treatment, which would be sufficient for early syphilis infections, according to the WHO^28^; however, the CCT demonstrated clear improvement in completing the repeated injections over time, which are recommended for people with unknown duration or latent syphilis. This suggests that community mobilisation events can provide avenues to syphilis testing and immediate, free treatment for multiple marginalised populations, which is particularly important given that over 40% of people with acquired syphilis are potentially asymptomatic and may not seek testing.^29^

The results also suggest that free diagnostics and treatment may be sufficient for treatment that requires one healthcare contact in situations when the patient is already present; however, free diagnostics and screening alone may not be sufficient for supporting treatments that require new clinical visits for screening or repeated clinical visits for treatment. In such scenarios, CCTs may provide critical support to populations facing economic barriers. This finding is supported by behavioural economic theory, which posits that an economic incentive may be an effective strategy to increase adherence to treatment, especially among low-income populations. While CCTs may face challenges in resource constrained settings, CCTs to support treatment completion among patients with latent syphilis or syphilis of unknown duration may be cost-saving when compared to the social and medical costs of long-term health sequelae of untreated syphilis^30^ and congenital syphilis,^31^ as well as the cost of lost productivity associated with infection or sequelae.^32^ Additional research is needed to evaluate whether such a program is cost-saving relative to long term costs associated with complications. While there is no reported resistance to treatment by penicillin, CCTs to support completion of treatment regimens may be particularly important for reducing risk of resistance to macrolides and tetracycline treatments in settings where penicillin is not available or where patients may have allergies to penicillin.^29^ Given the widespread use of CCTs in social policies in the LAC region, integrating targeted CCTs in health programs, including community health programs, should be administratively relatively feasible as the administrative expertise exists within state agencies. Further, arguments for CCT programs for individuals diagnosed with syphilis would likely be well-received, given the history of CCTs as a widely accepted development and anti-poverty strategy in the region coupled with their potential cost-effectiveness and the public health benefits.

While the sample size is limited, these findings also demonstrate the feasibility of integrating health services for displaced people (refugees, migrants, and returnees) into services provided to the host community, as recommended by UNHCR and UNAIDS.^33^^,^^34^ Not only is this important for promoting the health of the community as a whole in border settings and in settings hosting large numbers of internationally and internally displaced people but it also ensures health equity for all groups, while mitigating risk of stigma associated with services that aim to address sexually transmitted infections in one subpopulation. Given the difficulty of screening and treating intimate partners in pendular or in transitory migration situations for syphilis,^12^ providing CCTs in integrated syphilis services may help to address transmission and reinfection among couples.

This study focused on individuals in low-income communities in a border context, with a high share of displaced people and high levels of population mobility. Very little is known about adherence to syphilis treatment among the general adult population in the region. However, we would expect that CCTs could have a similar effect in other contexts with high levels of displaced people in the region. In principle, we would also expect that a CCT works similarly for individuals diagnosed with syphilis in low-income and vulnerable communities more broadly. However, additional studies are needed to rigorously assess generalisability.

### Limitations

Study findings should be interpreted with consideration to study limitations. The CCT intervention was not randomised but was sequentially assigned during two of four phases of a public health program. Some demographic differences between the individuals assigned to CCT and control groups were noted, for example, in nationality. The decision not to randomise was based on ethical considerations and feasibility of conducting the study in a community setting. We cannot rule out that the difference in treatment completion is not due to differences in study participants across arm. However, we feel this decision likely reduced the greater risk of selection bias or contamination that could have occurred with randomisation. Further, the results of the regression models, which remained robust when controlling for multiple potential confounders, combined with the sensitivity analysis using propensity score matching suggest that CCT is an effective method for encouraging syphilis treatment completion, though we cannot rule out potential residual confounding. The CCT intervention was conducted within the context of a community health mobilisation project; thus, we do not know whether a similar effect would be observed in other settings. The CCT may have varying effects for diverse subgroups, such as by sex, gender, or sexual orientation; however, our trial was not powered for subgroup analyses and further exploration is needed. The quasi-experimental trial was also conducted in a border city and, while findings may be informative and transportable to other similar settings serving migrants and host communities, they may not be generalisable to other areas.

### Conclusion

The results of this quasi-experimental trial in a border city of Colombia suggest that CCT may be effective in supporting syphilis treatment over free diagnosis and treatment alone in vulnerable populations, including displaced populations, who may otherwise face other indirect financial barriers to healthcare. These findings also underscore the feasibility of integrating internationally and internally displaced populations into innovative health programs provided for the host community.

## Contributors

MB: conceptualisation, study design, methodology, writing, literature review, project administration, supervision; ALW: study design, literature review, methodology, data analysis, data interpretation, writing; MP: study design, project administration, data curation; RO: data analysis; DP: study design, review, supervision. All authors had full access to all the anonymised data in the study and the corresponding author had final responsibility for the decision to submit for publication.

## Data sharing statement

The anonymised data is available upon request from the corresponding author.

## Declaration of interests

Magaly Pedraza received consultancy fees for management of the pilot project. Rafael Olarte received consultancy fees for initial statistical analysis.

## Appendix A Supplementary data

Supplementary Figures and Tables

Translated summary
