# Supplementary material for: Conditional cash transfer interventions to support syphilis treatment in vulnerable populations: a quasi-experimental study among displaced and host communities in a border city of Colombia
Source: Lancet Reg Health Am. 2025 Nov 14;53:101301. doi: 10.1016/j.lana.2025.101301 (PMC12664043; doi:10.1016/j.lana.2025.101301)

Supplemental Appendix

Conditional cash transfer interventions to support syphilis treatment in vulnerable populations: A quasi-experimental study among displaced and host communities in a border city of Colombia

Table 1. Characteristics of community workshop participants in a border city of Colombia, stratified by laboratory-confirmed syphilis

	Laboratory-confirmed syphilis result in community program					
	Negative (n=1,636)		Positive (N=114)		Total (N=1,751)	
	n	Col %	n	Col %	n	Col %
**Median age**, years (IQR)	31	(23-43)	34	(25-46)	31	(23-43)
**Sex**						
Male	523	31.9	50	43.9	573	32.7
Female	1114	68.1	64	56.1	1178	67.3
**Nationality**						
Colombian	757	46.2	51	44.7	808	46.1
Venezuelan or other	880	53.8	63	55.3	943	53.9
**Migration profile**						
Host population	523	32	22	19.3	545	31.1
Returned Colombian	233	14.2	30	26.3	263	15
In transit or pendular	49	3	9	7.9	58	3.3
Migrant with plans to stay	831	50.8	53	46.5	884	50.5
**Affiliated with health insurance**						
No	966	59	88	77.2	1054	60.2
Yes	671	41	26	22.8	697	39.8
**Marital status**						
Married or civil union	566	34.6	41	36	607	34.7
Single or separated	1071	65.4	73	64	1144	65.3
**Highest education started or completed**						
No formal education	48	2.9	14	12.3	62	3.5
Primary School	629	38.4	44	38.6	673	38.4
Highschool	909	55.5	46	40.4	955	54.5
University, Technical School, Technology Degree	51	3.1	10	8.8	61	3.5

Table 2. Estimated relative risks of the effect of CCT compared to control on the completion of treatment of syphilis in a sample of people with syphilis diagnosis in a border city of Colombia, 2023 (N=114)

	Model 1				Model 2				Model 3				Model 4			
**Characteristic**	**RR**	**95%CI**		**p-value**	**aRR**	**95%CI**		**p-value**	**aRR**	**95%CI**		**p-value**	**aRR**	**95%CI**		**p-value**
CCT (vs. Control)	1.7	1.3	2.3	0.001	1.9	1.4	2.6	p<0.001	1.9	1.4	2.6	p<0.001	1.9	1.4	2.9	p<0.001
Age (continuous)					1.0	1.0	1.0	0.107	1.0	1.0	1.0	0.096	1.0	1.0	1.0	0.057
Nationality: Venezuelan or other (vs. Colombian)					1.1	0.9	1.3	0.558	1.1	0.8	1.4	0.503	1.1	0.9	1.3	0.421
Female sex (vs. Male)					--	--	--	--	--	--	--	--	1.0	0.8	1.2	0.965
Education: Highschool or above (vs. none or primary)					--	--	--	--	0.9	0.8	1.2	0.593	--	--	--	--

Notes: Completed treatment is defined as received three doses of benzathine penicillin G intramuscularly weekly; RR: relative risk; aRR: adjusted relative risk calculated from model that includes listed co-variates (i.e., Model 2 estimates the effect of CCT vs control, adjusting for age and nationality); RRs and aRRs were calculated using generalised linear models for binomial outcomes with robust variance estimation; -- variable not included in model.

Table 3 Comparison of syphilis treatment completion among participants assigned to CCT (n=50) in a quasi-experimental trial in a border city of Colombia, 2023

Characteristic	Total (n=50)		Incomplete treatment (n=11)		Complete treatment (n=39)		p-value
**Median age (IQR)**	41	(29-52)	46	(37-53)	38	(27-52)	0.322
	**n**	**%**	**n**	**%**	**n**	**%**	
**Sex**							0.500
Male	25	50.0	5	45.5	20	51.3	
Female	25	50.0	6	54.6	19	48.7	
**Nationality**							0.269
Colombian	30	60.0	8	72.7	22	56.4	
Venezuelan or other	20	40.0	3	27.3	17	43.6	
**Affiliated with health insurance**							0.270
No	29	58.0	5	45.5	24	61.5	
Yes	21	42.0	6	54.6	15	38.5	
**Marital status**							0.144
Married or civil union	13	26.0	1	9.1	12	30.8	
Single or separated	37	74.0	10	90.9	27	69.2	
**Highest education started or completed**							0.620
No formal education or primary	27	54.0	6	54.6	21	53.9	
Highschool or above	23	46.0	5	45.5	18	46.2	

Notes: Completed treatment is defined as received three doses of benzathine penicillin G intramuscularly weekly, incomplete treatment is <3 doses; p-value calculated using Fisher's exact test

Table 4. Average treatment effects comparing conditional cash transfer (CCT) to control estimated by propensity score matching by age, nationality, and education

	RD	[95% conf.	interval]	p-value
Avg. treatment effect: CCT vs Control	0.29	0.09	0.49	0.005
**Covariate balance summary**	**Standardized differences**		**Variance ratio**	
	**Unmatched**	**Matched**	**Unmatched**	**Matched**
Age	0.676	-0.035	1.467	0.9160
Nationality	-0.562	-0.123	1.093	1.0198
Education	-0.110	-0.035	0.999	0.9900

Figure 1. Box plot of balance across intervention arm before and after matching based on propensity score for the model including age, nationality, and education

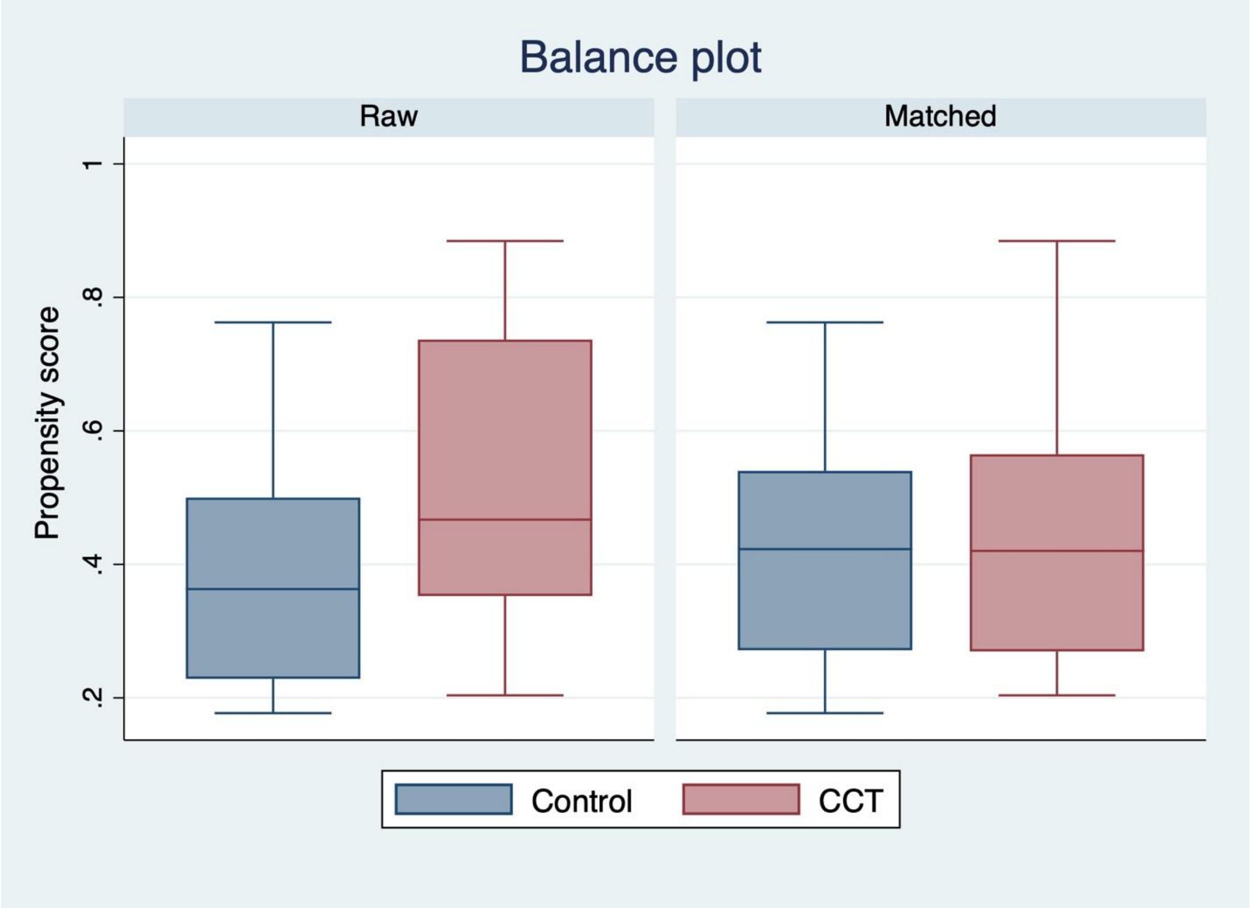

Table 5. Average treatment effects comparing conditional cash transfer (CCT) to control estimated by propensity score matching by age, nationality, education, and sex

	**RD**	**[95% conf.**	**interval]**	**p-value**
Avg. treatment effect: CCT vs Control	0.29	0.11	0.46	0.002
**Covariate balance summary**	**Standardized differences**		**Variance ratio**	
	**Unmatched**	**Matched**	**Unmatched**	**Matched**
Sex	-0.219	-0.147	1.054	1.096
Age	0.676	-0.055	1.467	0.859
Nationality	-0.562	-0.176	1.093	1.032
Education	-0.110	0.193	0.999	1.010

Figure 2. Box plot of balance across intervention arm before and after matching based on propensity score for the model including sex, age, nationality, and education

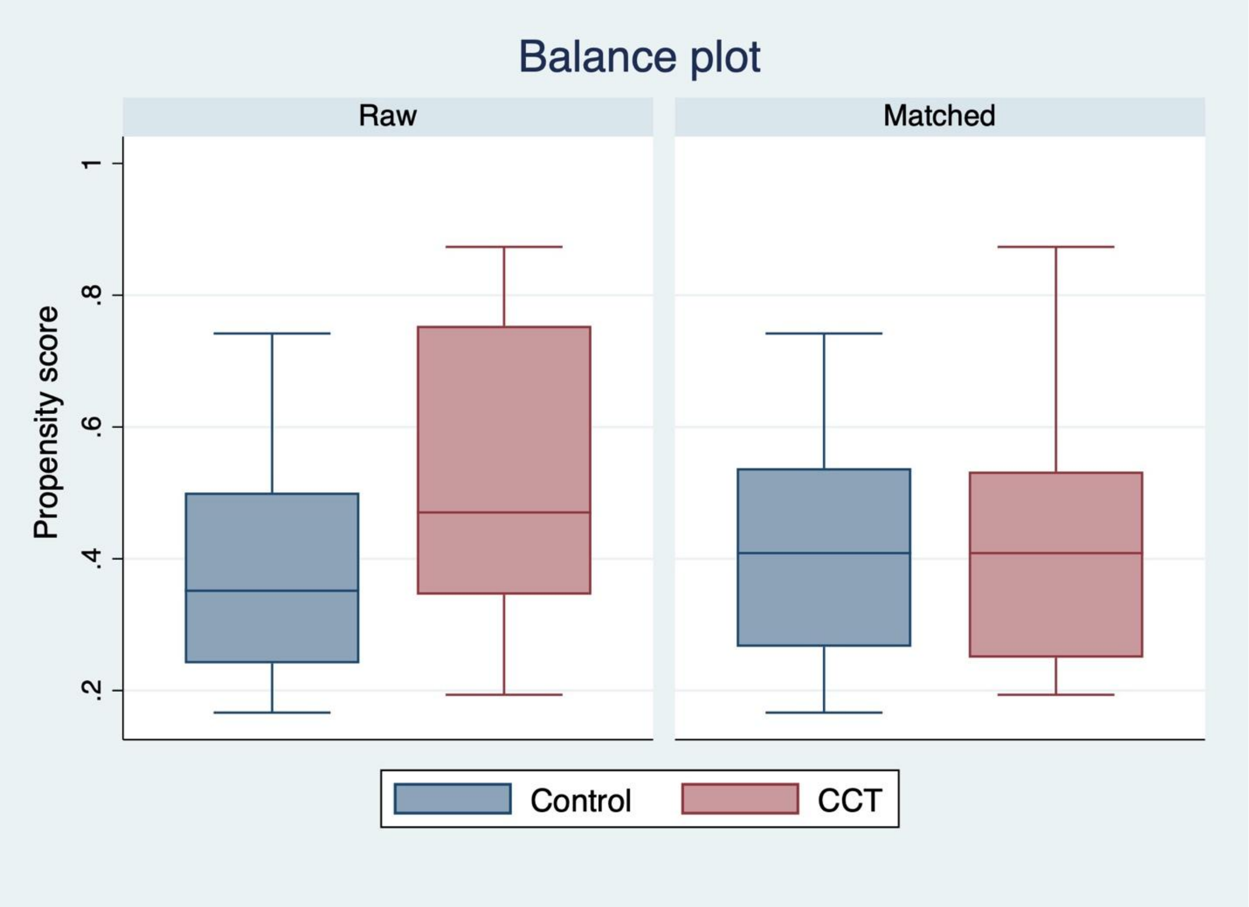

Supplement: Supplementary Figures and Tables [file mmc1.pdf]
