# Supplementary material for: Conditional cash transfer interventions to support syphilis treatment in vulnerable populations: a quasi-experimental study among displaced and host communities in a border city of Colombia
Source: Lancet Reg Health Am. 2025 Nov 14;53:101301. doi: 10.1016/j.lana.2025.101301 (PMC12664043; doi:10.1016/j.lana.2025.101301)
Supplement: Translated summary [file mmc2.pdf]

Editorial disclaimer: *This translation in Spanish was submitted by the authors and we reproduce it as supplied. It has not been peer reviewed. Our editorial processes have only been applied to the original abstract in English, which should serve as a reference for this manuscript.*

Resumen:

Antecedentes: La incidencia de la sífilis está aumentando en todo el mundo; sin embargo, el costo y el tiempo son obstáculos importantes para la adherencia al tratamiento entre las poblaciones vulnerables, incluidas las poblaciones desplazadas y las comunidades de acogida. Con el fin de informar las estrategias de salud pública, nuestro objetivo fue comprobar si las transferencias monetarias condicionadas (TMC) aumentaban la adherencia al tratamiento contra la sífilis en una muestra de la comunidad en una ciudad fronteriza de Colombia.

Métodos: Incorporamos una intervención cuasi-experimental de las transferencias monetarias condicionadas (TMC) en un programa comunitario de salud sexual dirigido a participantes de 14 años o más en asentamientos de bajos ingresos en el alrededor de Cúcuta, Colombia, en 2023. El programa incluía talleres y pruebas rápidas de sífilis. Las personas con sífilis confirmada por laboratorio eran elegibles para participar en la intervención. Tanto el grupo de control como el de TMC recibieron asesoramiento y tratamiento gratuito. La TMC consistió en pagos en efectivo de 12,69 dólares estadounidenses por completar cada uno de los dos tratamientos de seguimiento. Utilizamos modelos lineales generalizados para estimar el efecto de la TMC en la adherencia al tratamiento, definido como tres dosis de penicilina.

Resultados: De los 1751 participantes en el taller, 114 tenían sífilis confirmada por laboratorio y se inscribieron en el estudio. El 56 % de los participantes eran mujeres (64/114) y el 44 % hombres (50/114), y 6 participantes (5,3 %) se identificaron como transgénero, independientemente de su sexo de nacimiento. Entre los participantes había un 47 % de migrantes venezolanos que residían en Cúcuta (53/114), un 26 % de colombianos retornados (30/114) y un 19 % de colombianos parte de la comunidad de acogida (22/114). No se recogieron datos sobre el origen étnico. La edad mediana are 34,5 años (IQR: 25,0-46,0). Más de tres cuartos (78 %, 39/50) de los participantes de la TMC completaron el régimen de tratamiento de tres dosis, en comparación con el 45 % (29/64) de los participantes del grupo de control, lo que supone una diferencia de riesgo del 33 % ($p < 0,001$). En los modelos ajustados, los participantes asignados a la TMC tuvieron una tasa de adherencia al tratamiento un 36 % mayor en comparación con los participantes asignados al control (diferencia de riesgo ajustada: aRD: 0,36, IC del 95 %: 0,19-0,53).

Interpretación: Las TMC podrían mejorar la adherencia al tratamiento de la sífilis entre las poblaciones que se enfrentan dificultades socioeconómicas.
